# Supplementary material for: AAV ablates neurogenesis in the adult murine hippocampus
Source: eLife. 2021 Jul 14;10:e59291. doi: 10.7554/eLife.59291 (PMC8331179; doi:10.7554/eLife.59291)
Supplement: Supplementary file 1. — Post hoc comparisons of the electroporation groups presented in Figure 3E,F following two-way ANOVA. *p<0.05, **p<0.01, ***p<0.001, n.s. = not significant. [file elife-59291-supp1.docx]

| Cell growth (% Confluence) | | | | | | | | | | |
| --- | --- | --- | --- | --- | --- | --- | --- | --- | --- | --- |
| Time (min) | PBS vs. SCR 1E6 | PBS vs. SCR 5E6 | PBS vs. ITR 1E6 | PBS vs. ITR 5E6 | SCR 5E6 vs. SCR 1E6 | ITR 1E6 vs. SCR 1E6 | ITR 5E6 vs. SCR 1E6 | ITR 1E6 vs. SCR 5E6 | ITR 5E6 vs. SCR 5E6 | ITR 5E6 vs. ITR 1E6 |
| 3 | n.s. | n.s. | n.s. | n.s. | n.s. | n.s. | n.s. | n.s. | n.s. | n.s. |
| 6 | n.s. | n.s. | n.s. | * | n.s. | n.s. | * | n.s. | n.s. | ** |
| 9 | n.s. | n.s. | n.s. | *** | n.s. | n.s. | ** | n.s. | * | *** |
| 12 | n.s. | n.s. | n.s. | *** | n.s. | n.s. | *** | n.s. | ** | *** |
| 15 | n.s. | n.s. | n.s. | *** | n.s. | n.s. | *** | n.s. | *** | *** |
| 18 | n.s. | n.s. | n.s. | *** | n.s. | n.s. | *** | n.s. | *** | *** |
| 21 | n.s. | n.s. | n.s. | *** | n.s. | n.s. | *** | * | *** | *** |
| 24 | n.s. | n.s. | n.s. | *** | n.s. | n.s. | *** | ** | *** | *** |
| 27 | n.s. | * | n.s. | *** | n.s. | n.s. | *** | ** | *** | *** |
| 30 | n.s. | ** | n.s. | *** | n.s. | n.s. | *** | *** | *** | *** |
| 33 | n.s. | ** | n.s. | *** | * | n.s. | *** | *** | *** | *** |
| 36 | n.s. | ** | n.s. | *** | * | n.s. | *** | *** | *** | *** |
| 39 | n.s. | * | n.s. | *** | ** | n.s. | *** | *** | *** | *** |
| 42 | n.s. | * | n.s. | *** | * | n.s. | *** | ** | *** | *** |
| 45 | n.s. | * | n.s. | *** | n.s. | n.s. | *** | n.s. | *** | *** |
| 48 | n.s. | *** | n.s. | *** | n.s. | n.s. | *** | n.s. | *** | *** |
| 51 | n.s. | ** | n.s. | *** | n.s. | n.s. | *** | n.s. | *** | *** |
| 54 | n.s. | n.s. | n.s. | *** | n.s. | n.s. | *** | n.s. | *** | *** |
| 57 | n.s. | n.s. | n.s. | *** | n.s. | n.s. | *** | n.s. | *** | *** |
| 60 | n.s. | n.s. | n.s. | *** | n.s. | n.s. | *** | n.s. | *** | *** |
| Cell death (Proportion Propidium Iodide+) | | | | | | | | | | |
| Time (min) | PBS vs. SCR 1E6 | PBS vs. SCR 5E6 | PBS vs. ITR 1E6 | PBS vs. ITR 5E6 | SCR 5E6 vs. SCR 1E6 | ITR 1E6 vs. SCR 1E6 | ITR 5E6 vs. SCR 1E6 | ITR 1E6 vs. SCR 5E6 | ITR 5E6 vs. SCR 5E6 | ITR 5E6 vs. ITR 1E6 |
| 3 | n.s. | n.s. | n.s. | *** | n.s. | n.s. | *** | n.s. | *** | *** |
| 6 | ** | *** | *** | *** | n.s. | * | *** | n.s. | *** | *** |
| 9 | *** | *** | *** | *** | n.s. | ** | *** | * | *** | *** |
| 12 | *** | *** | *** | *** | n.s. | * | *** | n.s. | *** | *** |
| 15 | *** | *** | *** | *** | n.s. | * | *** | ** | *** | *** |
| 18 | *** | *** | *** | *** | n.s. | n.s. | *** | n.s. | *** | *** |
| 21 | ** | *** | *** | *** | n.s. | n.s. | *** | n.s. | *** | *** |
| 24 | * | *** | *** | *** | n.s. | n.s. | *** | n.s. | *** | *** |
| 27 | n.s. | *** | *** | *** | n.s. | n.s. | *** | n.s. | *** | *** |
| 30 | n.s. | ** | *** | *** | n.s. | n.s. | *** | n.s. | *** | *** |
| 33 | n.s. | ** | *** | *** | n.s. | n.s. | *** | n.s. | *** | *** |
| 36 | n.s. | * | ** | *** | n.s. | n.s. | *** | n.s. | *** | *** |
| 39 | n.s. | n.s. | ** | *** | n.s. | * | *** | n.s. | *** | *** |
| 42 | n.s. | n.s. | *** | *** | n.s. | * | *** | n.s. | *** | *** |
| 45 | n.s. | n.s. | *** | *** | n.s. | * | *** | * | *** | *** |
| 48 | * | n.s. | *** | *** | n.s. | n.s. | *** | ** | *** | *** |
| 51 | ** | n.s. | *** | *** | n.s. | n.s. | *** | *** | *** | *** |
| 54 | * | n.s. | *** | *** | n.s. | n.s. | *** | *** | *** | *** |
| 57 | * | n.s. | *** | *** | n.s. | n.s. | *** | *** | *** | *** |
| 60 | n.s. | n.s. | *** | *** | n.s. | n.s. | *** | ** | *** | *** |
